# CD13 Inhibition Enhances Cytotoxic Effect of Chemotherapy Agents

**DOI:** 10.3389/fphar.2018.01042

**Published:** 2018-09-12

**Authors:** Jian Zhang, Chunyan Fang, Meihua Qu, Huina Wu, Xuejuan Wang, Hongan Zhang, Hui Ma, Zhaolin Zhang, Yongxue Huang, Lihong Shi, Shujuan Liang, Zhiqin Gao, Weiguo Song, Xuejian Wang

**Affiliations:** ^1^School of Pharmacy, Weifang Medical University, Weifang, China; ^2^Weifang Bochuang International Biological Medicinal Institute, Weifang, China; ^3^School of Clinical Medicine, Weifang Medical University, Weifang, China; ^4^School of Bioscience and Technology, Weifang Medical University, Weifang, China

**Keywords:** CD13, MDR, bestatin, BC-02, 5FU

## Abstract

Multidrug resistance (MDR) of hepatocellular carcinoma is a serious problem. Although CD13 is a biomarker in human liver cancer stem cells, the relationship between CD13 and MDR remains uncertain. This study uses liver cancer cell model to understand the role of CD13 in enhancing the cytotoxic effect of chemotherapy agents. Cytotoxic agents can induce CD13 expression. CD13 inhibitor, bestatin, enhances the antitumor effect of cytotoxic agents. Meanwhile, CD13-targeting siRNA and neutralizing antibody can enhance the cytotoxic effect of 5-fluorouracil (5FU). CD13 overexpression increases cell survival upon cytotoxic agents treatment, while the knockdown of CD13 causes hypersensitivity of cells to cytotoxic agents treatment. Mechanistically, the inhibition of CD13 leads to the increase of cellular reactive oxygen species (ROS). BC-02 is a novel mutual prodrug (hybrid drug) of bestatin and 5FU. Notably, BC-02 can inhibit cellular activity in both parental and drug-resistant cells, accompanied with significantly increased ROS level. Moreover, the survival time of Kunming mice bearing H22 cells under BC-02 treatment is comparable to the capecitabine treatment at maximum dosage. These data implicate a therapeutic method to reverse MDR by targeting CD13, and indicate that BC-02 is a potent antitumor compound.

## Introduction

Hepatocellular carcinoma (HCC) is the fifth most common cancer type and the third leading cause of cancer-related deaths worldwide (Mlynarsky et al., 2015). Prognosis remains poor due to the low percentage of patients with HCC eligible for surgery (9–29%) (Tsurusaki and Murakami, 2015), high tumor recurrence rates after resection (60%) (Cheng et al., 2005), and limited benefit of conventional chemotherapy (Cao et al., 2012; Deng et al., 2015). The resistance of cancer cells to structurally and mechanistically unrelated classes of anticancer drugs is known as multidrug resistance (MDR) (Gottesman et al., 2002). And MDR is one of the major causes of chemotherapeutic failure in HCC therapy. Therefore, exploring more effective therapeutic strategies for patients with HCC is urgently needed. Increasing clinical trials have proposed that combination therapy may provide new strategy for chemo-resistance in patients with advanced HCC (Alves et al., 2011; Cervello et al., 2012; Shin and Chung, 2013).

Aminopeptidase N (APN, EC 3.4.11.2), which is also known as CD13, is a type 2 transmembrane Zn-dependent metallopeptidase of the gluzincin superfamily. APN forms a non-covalent bond homodimer on the cellular membrane. It hydrolyzes oligopeptides and releases neutral amino acids from the N-terminal end of small peptides. In human non-small cell lung cancer, pancreatic carcinoma, and prostate cancer, CD13 expression is associated with poor prognosis, and CD13 expression is involved in cancer invasion and metastasis (Tokuhara et al., 2006; Su et al., 2012). CD13 is also a marker for semi-quiescent cancer stem cells (CSCs) in human liver cancer cell lines and clinical liver cancer samples (Haraguchi et al., 2010). CSCs or tumor-initiating cells are responsible for drug resistance and tumor recurrence. CSCs express high level of ATP-binding cassette (ABC) transporters. Suppression of Pim-3 kinase expression by targeting CD13 can reverse MDR in HCC cells. Therefore, ABC transporters and Pim-3 may contribute to CD13 mediated HCC MDR (Guo et al., 2017).

Bestatin, which is a [(2S,3R)-3-amino-2-hydroxy-4-phenylbutanoyl] leucine obtained from the culture filtrates of *Streptomyces olivoreticuli*, is a dipeptide with low molecular mass. It is also a potent competitive inhibitor of CD13 with antitumor activity. Bestatin synergistically enhances the antitumor effects of anticancer drugs in HCC cell lines, and the effects of bestatin are due to the increased intracellular reactive oxygen species (ROS) levels (Yamashita et al., 2016). Our previous data indicated that CD13 inhibitor 4cc synergizes the antitumor effects of 5-fluorouracil (5FU) on human liver cancer cells in a ROS-dependent manner. CD13-neutralizing antibody (clone WM15, CD13 Ab) can also significantly induce ROS production compared with control (Sun et al., 2015).

In the current study, we aim to understand the role of CD13 in MDR and evaluate the antitumor effect of BC-02, a novel mutual prodrug (hybrid drug) of bestatin and 5FU, which can be degraded into bestatin and 5FU (Dou et al., 2017), on drug-resistant tumor cells. CD13 inhibitor bestatin and neutralizing antibody can enhance the sensitivity of tumor cells to cytotoxic agents. CD13 overexpression or knockdown affects the sensitivity of cells to cytotoxic agents. Compound BC-02 can inhibit both parental and drug-resistance tumor cell proliferation more markedly than single treatment of bestatin, 5FU, or a combination of 5FU and bestatin. All together, this study may bring new strategy to reverse MDR in HCC cancer.

## Materials and Methods

### Cell Culture and Reagents

Human hepatocarcinoma cell line PLC/PRF/5, Huh7, H7402, HepG2, and human colon cancer cell HCT116 were maintained in RPMI-1640 supplemented with 10% fetal calf serum (FCS). Human alveolar epithelial cell line A549 was grown in Dulbecco modified Eagle medium supplemented with 10% FCS. The cells were incubated at 37°C in a humidified atmosphere containing 5% CO_2_. Lipofection 2000 was purchased from Invitrogen (Cat. 11668-019). siRNA was synthesized by Shanghai GenePharma. Bestatin (Cat. B8385), 5FU (Cat. F6627), and cisplatin (cis-DDP, Cat. P4394) were purchased from Sigma. Gemcitabine (GEM, Cat. G8970), Paclitaxel (PTX, Cat. SP8020), and doxorubicin (DOX, Cat. D8740) were purchased from Solarbio. BC-02 (12a) was synthesized by conjugating bestatin and 5FU as previously described (Jiang et al., 2018).

### PLC/PRF/5-5FU Cell Culture

Low dose of 5FU was added into the medium of PLC/PRF/5. When cells need digest and passage, 5FU was also added after cell attachment. For a long time of incubation, higher concentration of 5FU was added. Then cells could survive at 40 μM 5FU.

### Flow Cytometry

Determination of CD13 expression by FACS was described previously (Wang et al., 2011). 1 × 10^5^ cells were washed with cold PBS and incubated with PE-conjugated monoclonal antibody targeting CD13 (BD Pharmingen, CD13mAb clone: WM15) for 60 min on ice. Then, the cells were analyzed on FACScan (FACSAria II; Becton-Dickinson). For ROS assay, cells were seeded and exposed to different drug samples. After 5 h incubation, cells were isolated and incubated at 37°C for 30 min with 10 μM 2,7-dichlorofluorescein diacetate (DCFH-DA) in the dark. Then the samples were washed and analyzed on a FACSCan.

### Cell Viability Assays

2 × 10^3^ cells/well were seeded in 96-well plate and allowed to grow for 4 h and the drugs were added to the wells at various concentrations. After 48 h, cells were incubated with 1% of 0.5 mg/ml MTT reagent for an additional 4 h. After that, the culture was removed, and the cells were lysed with 100 μl dimethyl sulfoxide (DMSO). The optical density of 570/630 nm was read on a plate reader (M5, MD) to calculate the inhibition rate. The inhibition rate of compounds was calculated by (ODcontrol-ODtested)/ODcontrol × 100%, where OD is the mean value of three replicate wells. The IC_50_ values were determined using ORIGIN 8 software (OriginLab Corporation, Northampton, MA, United States).

### Transfection Assay

Cells were seeded on a 96-well plate and transfected with siRNA targeting the sequence of CCGAAATGCCACACTGGTCAA of the human ANPEP (CD13) sequence (NM_001150) (Lai et al., 2012). Non-specific scrambled siRNA duplex was also purchased from GenePharma (Shanghai, China). The transfection protocol was according to the lipofection 2000 instruction.

### Lentivirus Infection

Lentivirus particles was supplied by GeneChem. The target of shRNA lentivirus was CCGAAATGCCACACTGGTCAA of the human ANPEP (CD13) sequence (NM_001150). The human ANPEP (CD13) sequence (NM_001150) was inserted into the vector of overexpression lentivirus. CD13 overexpression and knockdown lentivirus all overexpress green fluorescent protein. The procedure was according to the instruction. In brief, lentivirus particles was added into the medium of cells. Twelve hours later, the medium was replaced with completed culture medium. Then puromycin treatment help to get the stably overexpression or knockdown cells.

### Clone Formation Assay

Cells were plated in 6 or 48-well plates for overnight. Then cells were treated with different compounds for about 7–10 days. When the cells grew to visible colonies (>50 cells) the medium was discarded, and the cells were fiand with paraformaldehyde and stained with 0.1% crystal violet. Then clones were counted under an optical microscope.

### Western Blot

Either 20 or 30 μg of total protein of each lysate were subjected to 10 or 12% SDS–PAGE and electrotransferred onto PVDF membranes (Cat. IPVH00010, Millipore). Membrane was blocked with BSA and then incubated with primary antibodies. After washing, HRP-conjugated secondary antibodies were incubated. Washed with TBST, the bound antibodies were visualized by enhanced chemiluminescence (ECL, Cat. WBKLS0050, Millipore).

### *In vivo* Anti-tumor Assay

3 × 10^6^ H22 cells were injected to enterocoelia of Kunming mice. And mice were divided into different groups randomly and treated with agents. The survival period was recorded. For drug-resistant cell assay, H22-bearing KM mice were given 86 mg/kg/day capecitabine. After 2 weeks, tumor tissues were dissected from mice and triturated into single cell suspension. Then cells were implanted subcutaneously in KM mouse. Then mice randomized into vehicle and treatment groups, and mice were treated with BC-02 (130 mg/kg/day, ig) and capecitabine (370 mg/kg/day, ig). The mice body weight was monitored. After 2 weeks, all mice were sacrificed and dissected to weigh the tumor tissues. Animal experiment was approved by the Guidelines of the Animal Care and Use Committee of Weifang Medical University. The protocol was approved by the Animal Care and Use Committee of Weifang Medical University.

### Statistical Analysis

Data was presented as the mean ± SD, and analyzed by Student’s two-tailed *t*-test. The limit of statistical significance was *P* < 0.05. Statistical analysis was done with SPSS/Win11.0 software (SPSS Inc., Chicago, IL, United States).

## Results

### Cytotoxic Agent Results in Upregulation of CD13 Expression

**FIGURE 1:**
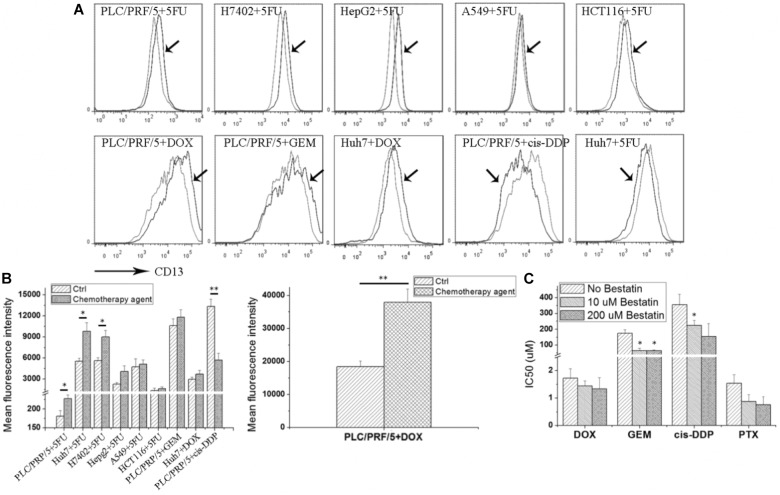
Cytotoxic agents increase CD13 expression, and CD13 inhibitor bestatin enhances the antitumor effect of cytotoxic agents. Different tumor cells were incubated with low cytotoxic agent dosage for 3 days, and CD13 expression was detected **(A)**. Geometric mean fluorescence intensity was shown **(B)**. MTT assay was employed to detect the viability inhibition after cytotoxic agent treatments combined with different bestatin concentrations **(C)**. Data represent mean ± SD (*n* = 3). ^∗^*P* < 0.05 and ^∗∗^*P* < 0.01 vs. Ctrl.

As shown in **Figure 1A**, after the 5FU treatment, CD13 expression was upregulated in hepatoma tumor cells, such as PLC/PRF/5, Huh7, H7402, and HepG2. 5FU could also increase CD13 expression in human alveolar epithelial cell line A549 and human colon cancer cell HCT116. Other cytotoxic agents, such as DOX and GEM, could also increase CD13 expression in PLC/PRF/5 and Huh7 cells. Meanwhile, cis-DDP could decrease CD13 expression of in PLC/PRF/5 cells.

CD13 upregulation induced by cytotoxic agent treatments demonstrated that CD13 may contribute to cell resistance to anticancer drugs. We supposed that CD13 inhibitor should enhance the cytotoxic effect of these agents. Our data indicated that CD13 inhibitor bestatin could enhance the cytotoxic effect of DOX, GEM, cis-DDP, and PTX (**Figure 1B**). Combination of bestatin and cytotoxic agents remarkably inhibited the cell viability of PLC/PRF/5 cells, compared with single treatment of cytotoxic agents (**Figure 1C**). Thus, the increased CD13 expression may protect cells from cytotoxic agents, and CD13 inhibitor bestatin enhances the cytotoxic effect of anticancer drugs.

### CD13-Targeting siRNA and Neutralizing Antibody Increase the ROS Level and Inhibit Cell Viability

**FIGURE 2:**
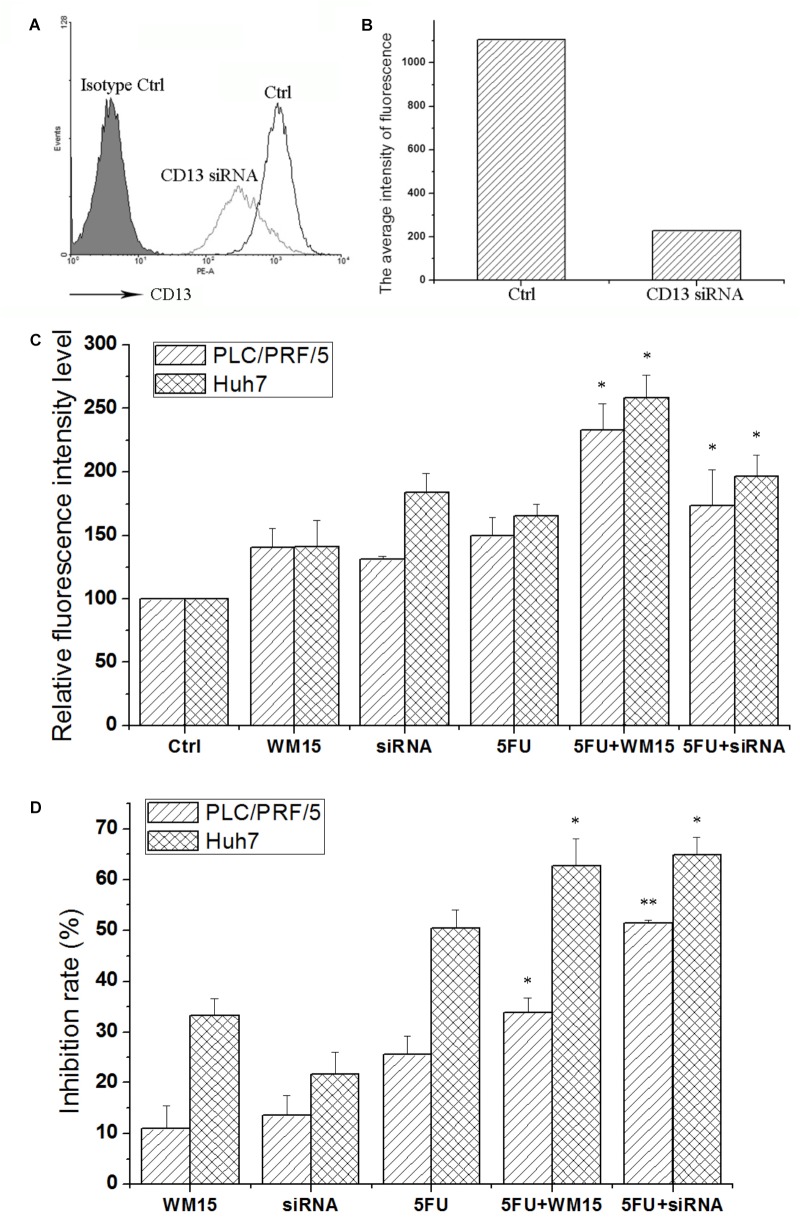
CD13 inhibition enhances the cytotoxic effect of 5FU. PLC/PRF/5 cells were transfected with CD13-targeting siRNA. FCS was used to detect CD13 expression **(A)**. **(B)** The average intensity of fluorescence of one experiment. The results were from a representative of at least three repeated experiments. PLC/PRF/5 and Huh7 cells were treated with CD13-neutralizing antibody, CD13-targeting siRNA, 5FU, a combination of neutralizing antibody and 5FU, and a combination of siRNA and 5FU. Then, ROS level **(C)** and cell viability **(D)** were detected. Data represent mean ± SD (*n* = 3). ^∗^*P* < 0.05 vs. 5FU, ^∗∗^*P* < 0.01 vs. 5FU. The transfection protocol was performed according to the instructions of lipofection 2000.

Although bestatin could enhance the cytotoxic effect of anticancer drugs, off-target effect for small molecular compound was observed. To certify the role of CD13 in protecting cells resistant to cytotoxic agent, CD13-targeting siRNA and neutralizing antibody were employed to suppress CD13. CD13-targeting siRNA could remarkably decrease CD13 expression (**Figures 2A,B**). siRNA and neutralizing antibody could also increase the ROS level in PLC/PRF/5 and Huh7 cells (**Figure 2C**). Compared with single 5FU, a combination of siRNA and neutralizing antibody with 5FU could remarkably increase the ROS level (**Figure 2C**). We also obtained similar result in MTT assay. Compared with single 5FU, siRNA and neutralizing antibody could remarkably enhance the inhibitory effect of 5FU on proliferation (**Figure 2D**). These data prove the importance of CD13 in tumor cell proliferation through the modulation of ROS generation.

### CD13 Overexpression Induces Cell Resistant to Cytotoxic Agent and CD13 Knockdown Leads to Sensitivity to Cytotoxic Agent

**FIGURE 3:**
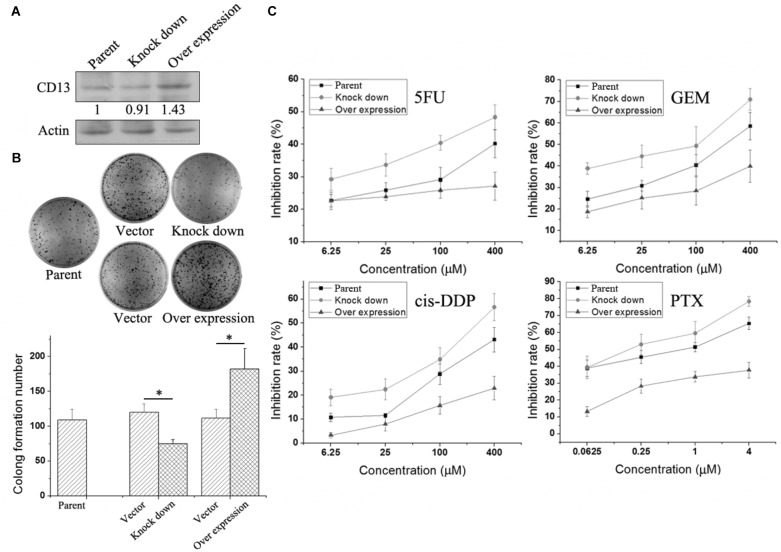
Effect of CD13 expression on drug resistance. PLC/PRF/5 cells were infected with lentivirus. After puromycin screening, PLC/PRF/5 cells with stable CD13 overexpression or knockdown were obtained **(A)**. A representative immunoblot from three independent experiments giving similar results is shown for each western blot experiment. Densitometry for western blot was performed using AlphaEaseFC-v4.0.0 program. 1 × 10^3^ PLC/PRF/5 cells, vector control, stable CD13 overexpression or knockdown PLC/PRF/5 cells were seeded in 6-well plates. Approximately 1 week later, cells were dyed with 0.1% crystal violet, and then photographs were taken **(B)**. The inhibition rate of different cytotoxic agents on overexpressed or knocked down cells were determined **(C)**. Data represent mean ± SD (*n* = 3). ^∗^*P* < 0.05.

To further verify the relationship between CD13 expression and drug resistance, we used a lentiviral vector to overexpress or knockdown CD13 expression. PLC/PRF/5 cells with stable CD13 overexpression or knockdown were obtained (**Figure 3A**). CD13 overexpression or knockdown could promote or inhibit cell colony formation (**Figure 3B**). Then, we detected the sensitivity of cells to cytotoxic agents. Compared with parental cells, CD13 overexpression induced cell resistance to 5FU, GEM, cis-DDP, and PTX (**Figure 3C**). In addition, CD13 knockdown sensitized cells to cytotoxic agents (**Figure 3C**).

### BC-02 Induces Higher ROS Generation Than 5FU and Inhibits Cell Viability

**FIGURE 4:**
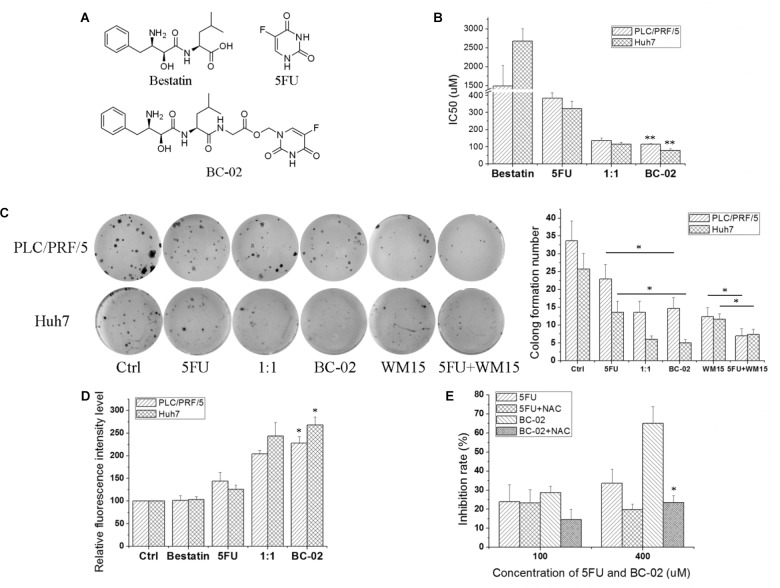
BC-02 increases ROS level and inhibits cell viability. **(A)** The chemical structure of compounds. PLC/PRF/5 and Huh7 cells were treated with bestatin, 5FU, equal bestatin and 5FU molars (1:1), and BC-02 for 48 h. Inhibition rate was determined using MTT assay, and IC50 value was calculated **(B)**. **(C)** 250 PLC/PRF/5 or Huh7 cells were seeded in 48-well plates. After 8 h, 2 μM drugs or 1 μg/ml neutralizing antibody were added. Approximately 1 week later, cells were dyed with 0.1% crystal violet, and then photographs were taken. **(D)** PLC/PRF/5 and Huh7 cells were treated with different drugs for 5 h, and ROS level was detected. **(E)** MTT assay was used to detect the inhibition rate of 5FU, 5FU+500 μM NAC, BC-02, and BC-02+500 μM NAC by using PLC/PRF/5 cells. Data represent mean ± SD (*n* = 3). ^∗^*P* < 0.05, ^∗∗^*P* < 0.01.

Compound BC-02 can be degraded into bestatin and 5FU (Dou et al., 2017). And BC-02 could inhibit the viability of PLC/PRF/5 and Huh7 cells more effectively, compared with single treatment of bestatin, 5FU, or a combination of 5FU and bestatin (**Figures 4A,B**). Clone formation assay also indicated that BC-02 could potently inhibit the clone formation of PLC/PRF/5 and Huh7 cells compared with 5FU and 1:1 combination group (**Figure 4C**). To verify specificity, we used CD13-neutralizing antibody, which could inhibit clone formation. Meanwhile, a combination of neutralizing antibody and 5FU could markedly inhibit clone formation compared with neutralizing antibody or 5FU alone (**Figure 4C**). Moreover, cellular ROS was detected by FCS. These data indicated that BC-02 could induce significantly higher level of ROS in PLC/PRF/5 and Huh7 cells more effectively, compared with single treatment of bestatin, 5FU, or a combination of 5FU and bestatin (**Figure 4D**). Moreover, ROS scavenger *N*-acetyl-L-cysteine (NAC) could protect cells from the cytotoxic effects of 5FU and BC-02 (**Figure 4E**). All these data together indicated that cell growth was inhibited through CD13 inhibition due to ROS generation.

### 5FU-Resistant Cancer Cells With Upregulated CD13 Expression Are More Sensitive to BC-02 Than 5FU

**FIGURE 5:**
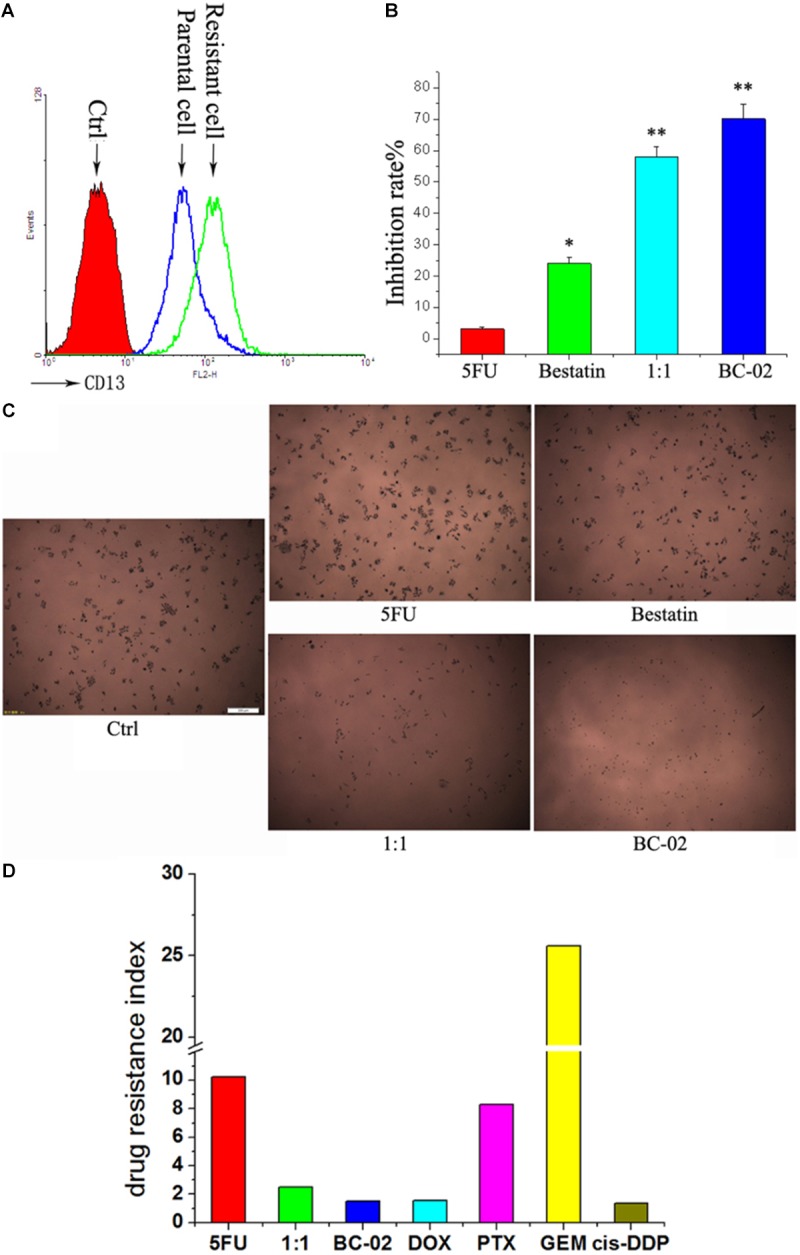
BC-02 inhibits the viability of 5FU-resistant cells. After long period of 5FU incubation, 5FU-resistance PLC/PRF/5 cells (PLC/PRF/5-5FU) can survive at 40 μM 5FU. CD13 expression was detected by FCS **(A)**. The inhibition rate of different drugs at a concentration 100 μM on PLC/PRF/5-5FU cells were determined **(B)**. After MTT was added for 2 h, photographs of cells were taken **(C)**. The IC50 values of different cytotoxic agents on PLC/PRF/5 and PLC/PRF/5-5FU cells were determined using MTT assay. In addition, drug resistance index was calculated using the IC50 value of PLC/PRF/5-5FU cells versus the IC50 value of PLC/PRF/5 cells **(D)**. Data represent mean ± SD (*n* = 3). ^∗^*P* < 0.05, ^∗∗^*P* < 0.01 vs. 5FU.

It is common to meet chemo-resistance for patients with HCC. Whether the chemo-resistant cells overexpress CD13 and remain sensitive to BC-02? To uncover this problem, we established 5FU-resistant PLC/PRF/5 cells (PLC/PRF/5-5FU) through low dose of 5FU incubation. After a long duration time of incubation with 5FU, PLC/PRF/5-5FU cells could survive at a concentration of 40 μM 5FU. FCS data confirmed that CD13 expression was upregulated in PLC/PRF/5-5FU chemo-resistant cells (**Figure 5A**). Moreover, PLC/PRF/5-5FU cells were resistant to 5FU but sensitive to BC-02 after being treated with 100 μM of either 5FU, bestatin, 5FU+bestatin (1:1), or BC-02 (**Figure 5B**). Photographs were also taken after MTT was added (**Figure 5C**). Almost no cells were observed in the BC-02 group. MTT assay further confirmed that almost no 5FU resistant cancer cells could survive after BC-02 treatment. The IC50 values of different cytotoxic agents to PLC/PRF/5 and PLC/PRF/5-5FU cells were determined, and drug resistance index was calculated using the IC50 value of PLC/PRF/5-5FU cells versus the IC50 value of PLC/PRF/5 cells. PLC/PRF/5-5FU cells were resistant to 5FU, PTX, and GEM, which were sensitive to BC-02, DOX, and cis-DDP, respectively (**Figure 5D**). All these data indicated that both parental and 5FU resistant cancer cells remain sensitive to BC-02.

### BC-02 Inhibits H22 Tumor Growth *in vivo*

**FIGURE 6:**
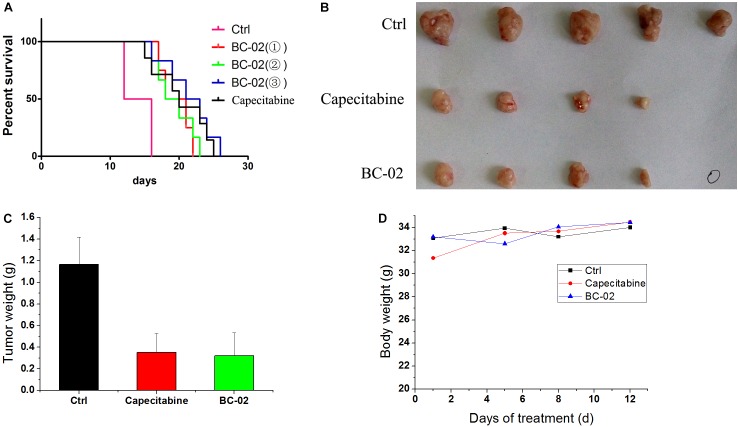
Antitumor activities of BC-02 *in vivo*. 3 × 10^6^ H22 cells were injected to the enterocoelia of Kunming mice. Then, mice were divided into different groups and treated with BC-02 and capecitabine, and survival period was recorded **(A)**. Capecitabine-resistant H22 cells was inoculated subcutaneously in KM mice, and they were treated with BC-02 and capecitabine. Then, mice body weight was monitored **(D)**. After 2 weeks, all mice were sacrificed and dissected to weigh tumor tissues **(B,C)**. “
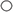
” means no tumor was found.

Capecitabine, a prodrug of 5FU, is used as a first- and second-line drugs for HCC treatment by several clinical trials (Murer et al., 2016; Casadei Gardini et al., 2017). The *in vivo* antitumor activity of capecitabine was stronger than that of 5FU in H22 cell-bearing Kunming (KM) mice transplant model (data not shown). Therefore, capecitabine was chosen as the positive control for our study in antitumor activity evaluation *in vivo*. In lifespan extension assay, H22 cell-bearing KM mice were treated with capecitabine (1 mmol/kg/day, iv), BC-02(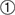) (0.15 mmol/kg/day, iv), BC-02(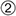) (0.075 mmol/kg, bid, iv), or BC-02(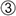) (0.1125 mmol/kg, bid, iv). Both BC-02 and capecitabine could extend the lifespan of mice, while BC-02(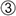) (0.1125 mmol/kg, bid, iv) was more potent than capecitabine (**Figure 6A**).

We also detected whether BC-02 inhibit the growth of capecitabine-resistant H22 cell. As described in the method section, capecitabine-resistant H22 cells were implanted subcutaneously in KM mice, and they were treated with BC-02 (130 mg/kg/day, ig) or capecitabine (370 mg/kg/day, ig). Both BC-02 and capecitabine could inhibit tumor growth (**Figures 6B,C**). No decrease in body weight was observed indicating the safety of BC-02 (**Figure 6D**). All together, BC-02 showed potent anti-tumor activity comparable to capecitabine *in vivo*.

## Discussion

HCC accounts for 85–90% of all liver cancer (El-Serag, 2011; Chacko and Samanta, 2016). Only a small portion of patients with HCC are available for surgery due to delayed diagnosis (Diaz-Gonzalez et al., 2016; Grandhi et al., 2016; Llovet et al., 2016; Mazzanti et al., 2016; Mazzoccoli et al., 2016). Because of low response rate and high toxicity, many chemotherapy agents have limited usage and can only provide minimal benefit to the survival time of patients with HCC (Simonetti et al., 1997; Connell et al., 2016). In this study, we found that CD13 was a therapeutic target which can reverse tumor cell MDR. Through the inhibition of CD13 activity, bestatin could enhance the cytotoxic effects of 5FU and other chemotherapy agents. Therefore, bestatin can be used as a good strategy for tumor therapy.

CD13 is a biomarker in human liver CSCs (Haraguchi et al., 2010), which are related to cancer MDR, recurrence, and metastasis. Therefore, we detected the relationship between CD13 and MDR. The results showed that CD13 inhibitor bestatin, CD13-neutralizing antibody, and CD13-targeting siRNA all could enhance the cytotoxic effect of 5FU and other chemotherapy agents. CD13 overexpression in PLC/PRF/5 cells could cause resistance to chemotherapy agents, while knocking down of CD13 could make PLC/PRF/5 cells to became sensitive to chemotherapy agents. All of these data together indicated that CD13 is a good therapeutic target to reverse MDR.

CD13-neutralizing antibody and bestatin can increase the ROS level in CD13^+^CD90^-^ PLC/PRF/5 and CD13^+^CD133^+^ Huh7 CSCs (Haraguchi et al., 2010). Excess of ROS induces cytotoxicity and apoptosis of cancer cells. Our previous work also indicated that BC-02 impaires the properties of liver CSCs by targeting CD13 and upregulating the intracellular ROS and ROS-induced DNA damage (Dou et al., 2017). APN inhibitor 4cc also synergizes the antitumor effects of 5FU in human liver cancer cells via ROS-mediated drug resistance inhibition and concurrent activation of the mitochondrial pathways of apoptosis (Sun et al., 2015). Therefore, we detected the relationship between CD13 inhibition and ROS. FCS data indicated that CD13 inhibitor bestatin, CD13-neutralizing antibody, and CD13 targeting-siRNA all could enhance the ROS upregulation effect of 5FU in tumor cells. Therefore, through CD13 inhibition, tumor cells cannot resist the ROS upregulation effect of 5FU, thereby leading to proliferation inhibition. Gclm participates in ROS scavenger pathway and encodes the glutamate-cysteine ligase which catalyzes the rate-limiting synthesis step of glutathione (GSH). GSH works as a critical cellular anti-oxidant and reducing agent. Gclm is overexpressed in the CD13^+^CD90^-^ fraction in PLC/PRF/5 cells (Haraguchi et al., 2010). Therefore, CD13 may protect cells from excessive ROS through up-regulation of Gclm.

Capecitabine has been tested as first- and second-line treatments for HCC by some studies (Murer et al., 2016; Casadei Gardini et al., 2017), and its antitumor activity was higher than that of 5FU in the mice transplant model. In the present assay, the capecitabine dosage was the maximum endurable dosage, while BC-02 was used at a much lower dosage. When treated with equal dosage, BC-02 performed better than capecitabine (data not shown). Moreover, BC-02 (0.1125 mmol/kg, bid, iv) was also more potent than the maximum endurable capecitabine dosage in lifespan assay. Furthermore, BC-02 was also sensitive in capecitabine-resistant H22 model. BC-02 achieved its antitumor activity through ROS upregulation. Silver nanoparticles also increased ROS level and lead to cell apoptosis (Wei et al., 2015). If BC-02 can be made into silver nanoparticles, its antitumor activity will be strengthened.

Li et al. (2015) reports that combining 5FU and bestatin could enhance the anticancer activity of 5FU in human tumor-derived cell lines and an H22 tumor-bearing mouse model. The authors mainly focused on normal tumor cells. In this study, we further indicated that the inhibition of CD13 could reverse the resistance of HCC cells to 5FU. ROS up-regulation is involved in the CD13 suppression induced cell death. However, we didn’t detect the ROS generation and elimination molecular. Therefore, the underlying molecular mechanism is still unclear and needs further research.

## Conclusion

Our study revealed CD13 as a promising target to reverse MDR. Through CD13 inhibition, the cytotoxic effect of chemo-agents will be enhanced via ROS upregulation. By the release of bestatin and 5FU, BC-02 remained sensitive to resistant cells. Taken together, BC-02 can be developed as a potent chemotherapeutic agent for human liver cancer.

## Author Contributions

JZ and CF participated in most of the experiments, such as cell biology and molecular biology experiments. MQ, HW, and XjuW performed the FCS assay. HZ and HM performed the MTT assay. ZZ and YH performed the mice assay. LS performed the colon assay. SL and ZG directed the data analysis. XjiW and WS designed the project.

## Conflict of Interest Statement

The authors declare that the research was conducted in the absence of any commercial or financial relationships that could be construed as a potential conflict of interest.
